# Socioeconomic inequalities in low birth weight in South Asia: A comparative analysis using Demographic and Health Surveys

**DOI:** 10.1016/j.ssmph.2022.101248

**Published:** 2022-10-11

**Authors:** Nusrat Jahan Sathi, Benojir Ahammed, Khorshed Alam, Rubayyat Hashmi, Ka Yiu Lee, Syed Afroz Keramat

**Affiliations:** a Statistics Discipline, Science, Engineering and Technology (SET) School, Khulna University, Khulna, 9208, Bangladesh; b School of Business, University of Southern Queensland, Toowoomba, QLD, 4350, Australia; c Centre for Health Research, University of Southern Queensland, Australia; d Swedish Winter Sports Research Centre, Department of Health Sciences, Mid Sweden University, Östersund, Sweden; e Economics Discipline, Social Science School, Khulna University, Khulna, Bangladesh; f QUT Business School, Queensland University of Technology, Brisbane, Queensland, Australia; g Centre for Health Services Research, Faculty of Medicine, The University of Queensland, Australia

**Keywords:** Concentration index, Children, Low birth weight, South Asia

## Abstract

**Background:**

Low Birth Weight (LBW) continues to be a prominent universal cause of various short- and long-term health hazards throughout infancy and adulthood. However, no study has revealed the socioeconomic inequalities in LBW among South Asian countries. This study assesses the socioeconomic inequalities among under-five South Asian children with LBW.

**Methods:**

Secondary data were derived from six (Afghanistan, Bangladesh, India, Maldives, Nepal, and Pakistan) nationally representative South Asian Demographic and Health Surveys conducted between 2015 and 2021, and included 170,547 under-five years of age children. The study employed the concentration curve and concentration index to assess the socioeconomic inequalities of those with LBW. Additionally, mixed-effect logistic regression was applied to determine the factors associated with LBW.

**Results:**

A significant negative concentration index indicates the wealth-related and education-related inequalities of LBW among under-five South Asian children. LBW is highly concentrated in the socio-economically poor section of the society. Our study found statistically significant negative concentration index in all South Asian countries: Afghanistan (Education: -0.108), Bangladesh (wealth: -0.070 & education: -0.083), India (wealth: -0.059 & education: -0.052), Nepal (by wealth: -0.064 & by education: -0.080), and Pakistan (by wealth: -0.080 & by education: -0.095). Findings from the mixed-effects logistic regression model also show that children from the poorest quintiles (AOR: 1.53, 95% CI: 1.41–1.67) and illiterate mothers (AOR: 1.39, 95% CI: 1.29–1.51) had higher odds of being afflicted with LBW compared to the wealthiest quintiles and educated mothers respectively. Women's pregnancy assessments, such as antenatal care utilisation, iron supplementation intake, and normal delivery mode, are significantly correlated with decreased odds of children's LBW.

**Conclusion:**

There exists a strong association between LBW cases and socioeconomic inequalities among South-Asian children below five years of age. This indicates the urgent need for health education and prenatal care services for women from Afghanistan, Bangladesh, India, Nepal, and Pakistan, especially those with lower socioeconomic status.

## Highlights

• Wealth- and education-related inequalities in low birth weight (LBW) are evident in South Asia.
• Afghanistan, Bangladesh, India, Nepal, and Pakistan have contributed significantly to the socioeconomic disparities in LBW in South Asia.
• Socioeconomic inequalities in LBW are higher in India compared to other countries in South Asia.

## 1 Introduction

Low Birth Weight (LBW) poses a public health threat since it contributes to several health complications during childhood and adulthood, including both acute and chronic disorders (Khan et al., 2018). The birth weight of a newborn baby less than 2500 g (<5.5 lbs.) is referred to as LBW (World Health Organization, 2006). It accounts for a 25 to 30-fold increased risk of infant mortality compared to standard birth weights (Tessema et al., 2021). The number of LBW cases is estimated to be 14.6 percent (20.5 million) worldwide, with the highest rates occurring in low and middle-income countries (LMICs) accounting for 91% of the total cases (Blencowe et al., 2019). Countries from the South Asian subcontinent register twice the number of LBW cases compared to Sub-Saharan African regions (Blencowe et al., 2019), with Bangladesh, India, and Pakistan having the largest contribution to the proportion of LBW children in the region (Leon & Moser, 2012). To lessen child mortality rates and attain the 2030 Sustainable Development Goals (SDGs) (Tessema et al., 2021, World Health Organization, 2014), the current number of cases needs to be reduced by 30% by 2025.

Socioeconomic Status (SES) is one of the contemporary measures that affect the number of LBW cases for each country. The well-being of children is directly related to maternal SES, as revealed by multiple studies conducted in several global settings (Jansen et al., 2009; Krieger et al., 2003; Mortensen, 2013; Reime et al., 2006). It was found that mothers from low-income neighbourhoods were more likely to have LBW babies. Studies also emphasised how disparities in educational attainment had a more substantial impact on childbirth outcomes, including LBW (Mortensen et al., 2008; Petersen et al., 2009). Children from the South Asian subcontinent face equivalent challenges (Abeywickrama et al., 2020; Mishra et al., 2021). Prolonged differences in SES destabilise societies, which might pose a severe danger to achieving the World Health Organization's (WHO) global nutritional targets for South Asia in the upcoming decades (South Asia Alliance for Poverty Eradication, 2019).

A wide range of studies on LBW and its associated factors from the South Asian context were observed in the literature (Ahammed et al., 2020; Badshah et al., 2008; Gupta et al., 2019; Khan et al., 2018). These investigations incorporated a range of internationally recognised and permitted data sources, including nationally representative surveys and hospital-based cross-sectional prospective information. Extant research has discovered that several maternal-infant behaviours and lifestyle-related indicators, including age, antenatal care (ANC) utilisation status, place of residence, birth order, delivery mode, and sex, substantially affect the rate of children affected by LBW. However, looking at the currently available literature, it is evident that no study has evaluated the SES inequalities of LBW in South Asia, despite the stark discrepancies in the continent (South Asia Alliance for Poverty Eradication, 2019). The incidence of LBW varies across the region, with South Asia exhibiting the highest cases of LBW in children (Mishra et al., 2021). SES is related to children's health outcomes like LBW, as Ross and Mirowsky (2008) discovered that SES had a substantial favourable impact on health (Ross & Mirowsky, 2008). The role of mother SES traits, such as wealth index and education, is pivotal to ensuring the well-being of children (Mishra et al., 2021). It is evident that South Asia could not achieve equal economic development across all regions, although it is required to achieve the SDGs (Rama et al., 2015; South Asia Alliance for Poverty Eradication, 2019). Identifying unequal SES development on LBW throughout the South Asian subcontinent is crucial for achieving the SDGs' goal of reducing child mortality. However, two studies from India and Sri Lanka investigated the SES inequalities of LBW on an individual basis (Abeywickrama et al., 2020; Mishra et al., 2021). Due to the two countries' specific nuance, these studies were restricted to demonstrating the aggregate scenario of SES inequalities considering LBW for the South Asian region. It is still an unexplored area for South Asia because no studies have examined SES disparities in LBW across South Asian subcontinents.

To address this gap in the literature, this article focuses on measuring SES inequalities in South Asia using a large-scale, nationally representative dataset. The study hypothesizes that children born into lower SES households are more prone to LBW than those born in higher SES households. The study also determines the prevalence and associated factors of LBW among under-five children in South Asia, with inconsistent output for several variables documented in studies from South Asian regions (Ahammed et al., 2020; Badshah et al., 2008; Gupta et al., 2019; Khan et al., 2018). Hence, this investigation appends a new dimension in the literature by generalising estimation over the South Asian setting. The findings may assist government officials and policymakers, and other stakeholders tasked to design health strategies considering SES aspects to control LBW.

## 2 Material and methods

### 2.1 Data source

This study was based on the secondary data sources driven from the most recent Demographic and Health Surveys (DHSs) from six (6) South Asian countries, including Afghanistan (2015), Bangladesh (2017–2018), India (2019–2021), Maldives (2016–2017), Nepal (2016), and Pakistan (2017–18). These datasets were merged to evaluate SES inequalities and investigate the most significant factors of LBW across the region. This nationally representative DHS program collected data on various health indicators, focusing on mother and child health. The survey programs followed a standardised sampling technique, ensuring that the survey design was identical across the countries (Croft et al., 2018). Bhutan and Sri Lanka are the other two South Asian countries that were excluded from the distribution of study participants because i) Bhutan is not listed in the DHS program, and ii) the most recent Sri Lankan DHS dataset is not available in the public domain.

**Fig. 1:**
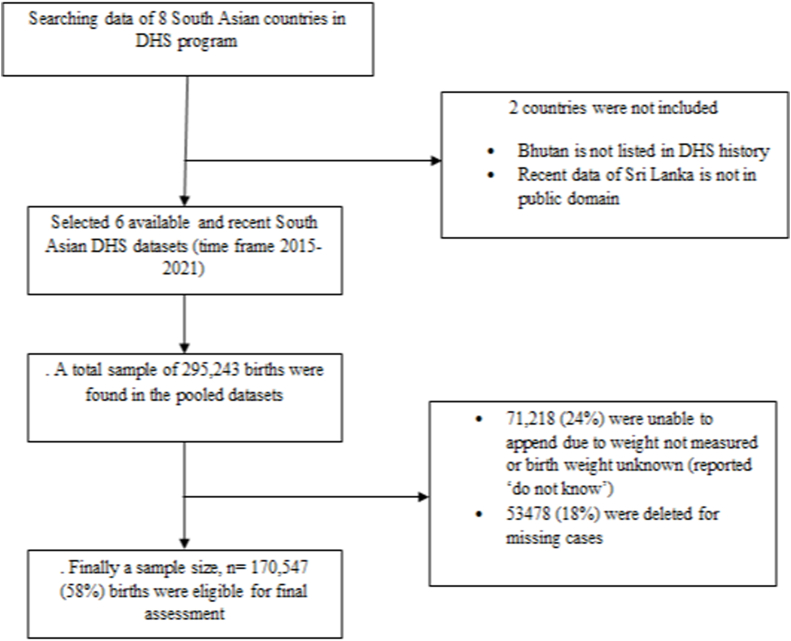
Description of study participants for LBW from South Asian regions utilising Demographic and Health Surveys.

The DHS selected study participants in two stages using a stratified sampling technique. The survey methodology and sampling techniques used to collect the data have been described in detail elsewhere (Central Statistics Organization, Ministry of Public Health, & ICF, 2017, International Institute for Population Sciences - IIPS India, ICF, 2017, Ministry of Health - MOH Maldives, ICF, 2018, Ministry of Health - MOH/Nepal, New ERA/Nepal, ICF, 2017, National Institute of Population Research and Training -NIPORT, Ministry of Health and Family Welfare, ICF, 2020, National Institute of Population Studies - NIPS/Pakistan, ICF, 2019). The DHS maintains various data files, including men, women, children, birth, and household. The study utilised Kids Record (KR) files that contain data on all newborns under the age of five delivered during the past five years prior to the surveys. By merging these six DHS datasets from South Asian subcontinents, a total of 170,547 under-five children were appended in the final selection (Fig. 1).

### 2.2 Variables of the study

#### 2.2.1 Dependent variable

Low Birth Weight was the main response variable used in this study. Women who gave birth during the last five years before the recent survey were eligible to provide weight data for each country in South Asia. They reported birth weights for their children based on record assessment or reminiscence. The study then eliminated observations that had not documented birth weight information. Following conventional definition, LWB was defined as a birth weight of less than 2500 g (g). In contrast, normal or above-normal birth weights were classified as 2500 g or above (World Health Organization, 2006). Therefore, the LBW variable is binary, where ‘1’ was referred to <2500 g, and otherwise, it was coded as ‘0’.

#### 2.2.2 Independent variables

Existing literature greatly helped in the inclusion of probable LBW factors for the current study (Badshah et al., 2008; Dharmalingam et al., 2009; Gupta et al., 2019; Khan et al., 2018; Long et al., 2012; Mberu et al., 2016; Mishra et al., 2021; Tessema et al., 2021). The study considered variables at two levels: individual-level variables (Level-1) and community-level variables (Level-2). One on hand, Level-1 includes the mother's age in years (≤19, 20–24, 25–29, and 30 or above), mother's educational attainment (no education, primary, secondary, and higher), wealth index (poorest, poorer, middle, richer, and richest), sex of child (male and female), birth status (singleton and multiple), ANC visit (no, 1 to 3, and 4 or above), iron tablets/syrup intake during pregnancy (no and yes), and mode of birth (normal and caesarean section). On the other hand, Level 2 consists of residential status (rural and urban). The mother's age and the number of ANC visits were continuous variables that were transformed into categorical variables before final statistical execution. The wealth index quintiles were constructed using principal component analysis on the cumulative living standards of households. Singleton is a category of birth status that describes the birth of a single infant, whereas “multiple” refers to an infant born in more than one birth case.

### 2.3 Data management and analysis

This study analysed the available data using various statistical techniques. Prior to that, sampling weights, primary sampling units, and strata were evaluated to adjust the complex survey design. At the outset, cross-tabulation was deployed to depict the distribution of baseline characteristics through STATA version 16 software. A fixed-effect meta-analysis showed LBW cases' prevalence in South Asia and its sub-regions. A forest plot highlighted the prevalence with 95% confidence intervals.

Standard logistics and mixed-effect logistic regression models were used in the multivariate stage. Statistical parameters were reported to find the best-fitted model, including log-likelihood, Akaike information criterion (AIC), and Bayesian information criterion (BIC). The lowest magnitude of the AIC and BIC was selected to identify the associated factors of LBW cases. The suitable model for the dataset was the mixed-effect logistic regression model. The underlying explanation was that observations were nested within clusters due to the hierarchical structure of DHS data. This hierarchical nature violates the observations' independence and equal variance assumption of the traditional logistics model. The random part of the mixed-effect logistic regression model was appended in the main findings: Likelihood Ratio (LR) test, cluster variance, Intra-class Correlation Coefficient (ICC), and Median Odds Ratio (MOR). The estimated value of MOR is greater than one, implying a cluster effect on the outcome. In contrast, an estimated value of MOR equals one means an absence of cluster disparities (Merlo et al., 2005). Adjusted Odds Ratio (AOR) was included, where AOR, 95% confidence interval (CI), and P-value were utilised to identify the substantially associated predictors of LBW in the region.

In addition, both Concentration Index (CI) and Concentration Curve (CC) were applied to assess SES inequalities according to wealth and education levels. The cumulative share of LBW cases was plotted against the cumulative percentage of children, ranked according to wealth index (poorest to richest) and educational attainment (lowest to highest) (O'Donnell et al., 2008). The CC expressed three conclusions about its stance on the 45-degree line of equality: pro-poor inequalities (above), pro-rich inequalities (below), and no inequalities (on the 45-degree line). CI is deployed to measure the magnitude of SES inequalities over the CC, as CC only displays the disparities. In general, CI refers to twice the region between the CC and the line of equality (Konings et al., 2009), and the value lies between −1 and +1. A negative value indicates that the CC is above the line of equality. In contrast, a positive value indicates that the CC is below the line of equality, and a zero indicates that the CC is on the line of equality. The upper and lower bound of CI might not be between −1 and +1 due to the binary nature of the outcome (Wagstaff, 2005). The standard CI accounts for the mean of the binary outcome, which influences the upper and lower boundaries (Ataguba, 2022). To adjust for these shortcomings (Pulok et al., 2018), three methodologies were used to evaluate the SES inequalities: Standard, Wagstaff, and Erreygers. Wagstaff CI and Erreygers CI are two rescaling approaches to bound the CI limits into [−1, +1] in case of the binary outcome, considering the invariant property (Erreygers, 2009; Wagstaff, 2005). The outputs include index values, robust standard errors, P-values, and 95% confidence intervals.

Decomposition technique (both Wagstaff and Blinder Oaxaca) utilised ordinary least squares regression methods to reveal the contribution of different explanatory variables while explaining SES inequalities (Samuel et al., 2021; Wagstaff et al., 2003). The outcome variable of the present study is binary and we used mixed-effects logistic regression technique as per our designed technique. Therefore, we cannot perform decomposition technique to see the contribution of different factors on the socioeconomic inequality in LBW. Performing decomposition technique will result in bias estimates as the decomposition techniques is based on the OLS technique.

## 3 Results

### 3.1 Summary statistic of background characteristics

Table 1 (shown below) represents the percentage distribution of LBWs among children regarding background characteristics from South Asian countries. The highest occurrence of LBW among under-five children was observed in women who gave birth at very early ages (22.02%).

**Table 1:** Background characteristics of the selected sample from the South Asian Demographic Health Survey (DHS) program.

Characteristics	Total		Low Birth Weight				*P*-value
			Un-weighted Prevalence		Weighted Prevalence		
	n	(%)	n	(%)	n	(%)	
**Mother age (years)**							<0.001
≤19	5240	3.07	1109	21.16	1284	22.02	
20–24	47,568	27.89	8767	18.43	9476	19.00	
25–29	64,937	38.08	10,587	16.30	11,006	16.88	
30 and above	52,802	30.96	7988	15.13	7904	16.15	
**Mother education**							<0.001
Higher	27,944	16.38	3692	13.68	4271	13.61	
Secondary	90,113	52.84	14,808	16.43	15,481	17.50	
Primary	20,553	12.05	3832	18.64	3984	20.11	
No education	31,937	18.73	6119	19.16	5934	19.68	
**Wealth index**							<0.001
Richest	27,629	16.20	3974	14.38	4698	14.43	
Richer	31,774	18.63	4887	15.38	5543	16.14	
Middle	34,536	20.25	5477	15.86	5766	16.97	
Poorer	38,184	22.39	6624	17.35	6596	18.97	
Poorest	38,424	22.53	7489	19.49	7067	20.69	
**Sex of child**							<0.001
Male	91,662	53.75	14,219	15.51	14,725	16.09	
Female	78,885	46.25	14,232	18.04	14,945	19.08	
**Birth Status**							<0.001
Singleton	168,926	99.05	27,489	16.27	28,606	17.01	
Multiple	1621	0.95	962	59.35	1064	63.18	
**ANC visit**							<0.001
No	8252	4.84	1649	19.98	1735	20.60	
1 to 3	57,095	33.48	10,228	17.91	10,369	18.72	
4 or above	105,200	61.68	16,574	15.75	17,566	16.57	
**Iron tablets/syrup intake during pregnancy**							<0.001
Yes	151,912	89.07	24,852	16.36	26,064	17.21	
No	18,635	10.93	3599	19.31	3606	19.58	
**Mode of birth**							0.793
Normal	130,673	76.62	21,782	16.67	21,948	17.42	
Caesarean section	39,874	23.38	6669	16.73	7722	17.60	
**Residential Status**							<0.001
Rural	130,122	76.30	22,085	16.97	21,216	17.91	
Urban	40,425	23.70	6366	15.75	8454	16.45	

Children from the lowest wealth quintiles (20.69%) and mothers with primary education (20.11%) had a greater probability of being afflicted with LWB. Female children (19.08%) and children from multiple births (63.18%), caesarean deliveries (17.60%), and rural residents (17.91%) reported a higher instance of LBW across the subcontinent. Additionally, mothers who did not take ANC services (20.60%) and iron tablets (19.58%) during the gestational period were accountable for higher LBW incidence among South Asian children.

### 3.2 Prevalence of LBW in South Asia

**Fig. 2:**
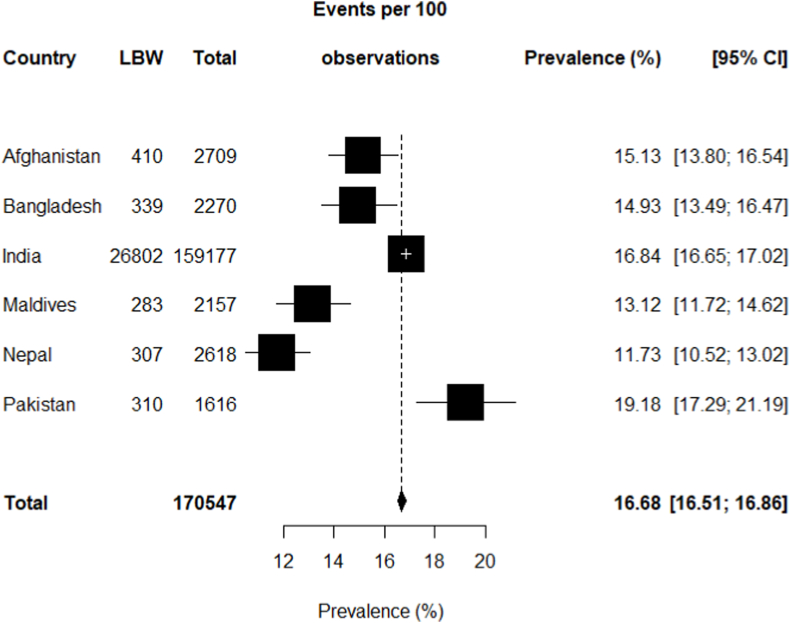
Forest plot of prevalence of LBW among South Asian under-five children.

Fig. 2 shows the prevalence of LBW among South Asian children under-five through a forest plot. Among the 170,547 participants, 17% of children had LBW in South Asia. The reported information exhibits that the highest LBW weighted prevalence was in Pakistan (19.18%), followed by India (16.84%), Afghanistan (15.13%), Bangladesh (14.93%), and Maldives (13.12%), while the lowest LBW prevalence was in Nepal (11.73%).

### 3.3 Analysis of SES inequalities

Table 2, Table 3 (shown below) demonstrate wealth and education-related inequalities of LBW among children under-five in South Asian countries by three different concentration indices.

**Table 2:** Wealth-related inequalities of low birth weight among under-five children in South Asia.

*Country*	Standard Concentration Index			Wagstaff Concentration Index			Erreygers Concentration Index		
	Index Value (Robust SE)	95% CI		Index Value (Robust SE)	95% CI		Index Value (Robust SE)	95% CI	
*Afghanistan*	0.026 (0.066)	−0.103	0.154	0.031 (0.079)	−0.125	0.187	0.018 (0.046)	−0.072	0.107
*Bangladesh*	−0.070** (0.032)	−0.132	−0.008	−0.084** (0.038)	−0.158	−0.010	−0.046** (0.021)	−0.086	−0.005
*India*	−0.059*** (0.004)	−0.067	−0.051	−0.073*** (0.005)	−0.082	−0.063	−0.043*** (0.003)	−0.049	−0.037
*Maldives*	−0.054 (0.044)	−0.140	0.032	−0.062 (0.051)	−0.161	0.037	−0.028 (0.023)	−0.072	0.017
*Nepal*	−0.064** (0.030)	−0.122	−0.007	−0.074** (0.034)	−0.140	−0.007	−0.032** (0.015)	−0.060	−0.003
*Pakistan*	−0.080* (0.042)	−0.163	0.003	−0.102* (0.054)	−0.209	0.004	−0.070* (0.037)	−0.143	0.003
*Pooled*	−0.059*** (0.004)	−0.067	−0.050	−0.072*** (0.005)	−0.082	−0.061	−0.042*** (0.003)	−0.049	−0.036

Abbreviation:SE= Standard error; CI= Confidence interval. Significance level: *p < 0.10, **p < 0.05, and ***p < 0.01.

**Table 3:** Education-related inequalities of low birth weight among under-five children in South Asia.

*Country*	Standard Concentration Index			Wagstaff Concentration Index			Erreygers Concentration Index		
	Index Value (Robust SE)	95% CI		Index Value (Robust SE)	95% CI		Index Value (Robust SE)	95% CI	
*Afghanistan*	−0.108** (0.048)	−0.202	−0.015	−0.131** (0.058)	−0.244	−0.018	−0.075** (0.033)	−0.140	−0.010
*Bangladesh*	−0.083** (0.032)	−0.146	−0.019	−0.099** (0.039)	−0.174	−0.023	−0.054** (0.021)	−0.095	−0.013
*India*	−0.052*** (0.004)	−0.059	−0.045	−0.064*** (0.004)	−0.072	−0.055	−0.038*** (0.003)	−0.043	−0.033
*Maldives*	−0.060 (0.040)	−0.139	0.020	−0.068 (0.046)	−0.160	0.023	−0.031 (0.021)	−0.072	0.010
*Nepal*	−0.080** (0.033)	−0.144	−0.015	−0.091** (0.038)	−0.165	−0.017	−0.039** (0.016)	−0.071	−0.007
*Pakistan*	−0.095*** (0.036)	−0.165	−0.025	−0.122*** (0.046)	−0.211	−0.032	−0.083*** (0.031)	−0.145	−0.022
*Pooled*	−0.054*** (0.004)	−0.062	−0.046	−0.066*** (0.005)	−0.075	−0.056	−0.039*** (0.003)	−0.045	−0.033

Abbreviation: SE= Standard error; CI= Confidence interval. Significance level: *p < 0.10, **p < 0.05, and ***p < 0.01.

Inverse and statistically significant index values are discovered for pooled cases from all six countries (shown in Table 2, Table 3). In the pooled cases, the index values for wealth-level and education-level are −0.059 & −0.054 (Standard CI), −0.072 & −0.066 (Wagstaff CI), and −0.042 & −0.039 (Erreygers CI), respectively. All negative values suggest that LBW is more rampant among children from lower-income households and mothers with less education. India, Nepal, Bangladesh, and Pakistan provide statistically significant index values (Table 2). Except for the Maldives, all nations in Table 3 have a negative and statistically substantial concentration index. This indicates that the statistical significance of estimates from six countries derived from all three measures yields consistent conclusions.

All three measurements have negative magnitudes for significant index values; Erreygers CI has the highest index value, followed by Standard CI and Wagstaff CI (Table 2 & Table 3). However, Standard CI, Wagstaff CI, and Erreygers CI provide consistent magnitude. Children from India had the largest SES inequalities (according to wealth and education level) in LBW, followed by those from Nepal, Bangladesh, Afghanistan, and Pakistan. The CCs also support these outcomes of inequalities in education and income levels. Additionally, the findings indicate that wealth-related inequalities associated with LBW are higher in children than in education-related inequalities.

**Fig. 3:**
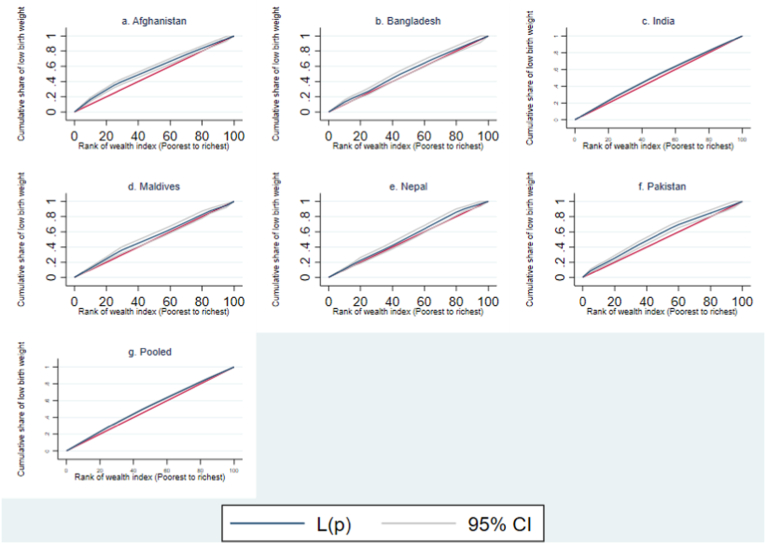
Concentration curve of low birth weight (by Standard concentration index with wealth index ranking).

**Fig. 4:**
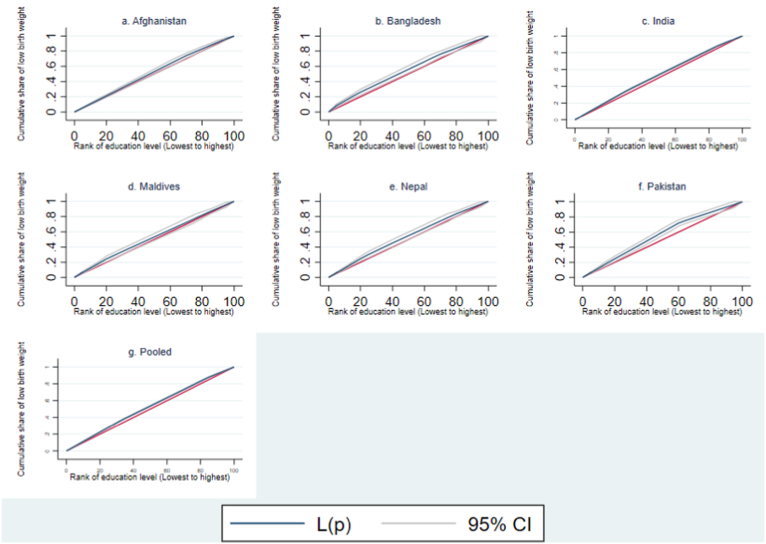
Concentration curve of low birth weight (by Standard concentration index with education level).

The CCs of LBW by wealth index and education level following the Standard Concentration Index (SCI) are depicted in Fig. 3, Fig. 4. The CCs are above the line of equality (45° line) for both cases. This indicates a higher LBW concentration towards children from the lowest wealth quintiles and mothers with less education. Among all six South Asian countries, the highest index value was found in India simultaneously regarding wealth index and education level. Figures A1-A4 in the additional file include the CCs for LBW by Wagstaff Concentration Index (WCI) and Erreygers Concentration Index (ECI), which uniformly correspond to the illustrated findings (see Additional file 1).

### 3.4 Regression analysis

Table 4 illustrates the findings of mixed-effects logistic regression for identifying individual-level and community-level factors associated with LBW among under-five children in South Asia. The value of Intra-class Correlation (ICC) from Model 0 reveals that indicators added at the community level account for roughly 15.21% of the variation in the LBW. This is enough evidence that mixed-effects logistics regression was the best fit over the standard logistics model as the magnitude of ICC is larger than zero. The significant outcomes also validated this agreement from the LR test. Moreover, a MOR of +2.01 implies that clustering has an impact on LBW cases, which supports conducting the analysis using mixed-effects logistics regression.

**Table 4:** Associated factors of LBW from mixed-effects logistic regression model among under-five children in South Asia.

Variables	Model 0^a^		Model 1^b^		Model 2^c^		Model 3^d^	
	**Odds Ratio**	**95% CI**	**Adjusted Odds Ratio**	**95% CI**	**Adjusted Odds Ratio**	**95% CI**	**Adjusted Odds Ratio**	**95% CI**
***Intercept***	**0.18**	**[0.18**–**0.19]**	**0.15**	**[0.13**–**0.17]**	**0.14**	**[0.11**–**0.18]**	**0.13**	**[0.02**–**0.17]**
**Individual-level variables**								
***Mother age (years)***								
≤*19 (Ref)*			1.00				1.00	
*20–24*			**0.82**	**[0.74**–**0.91]**			**0.82**	**[0.74**–**0.91]**
*25–29*			**0.72**	**[0.64**–**0.80]**			**0.72**	**[0.64**–**0.80]**
*30 and above*			**0.66**	**[0.59**–**0.73]**			**0.66**	**[0.59**–**0.73]**
***Mother Education***								
*Higher (Ref)*			1.00				1.00	
*Secondary*			**1.25**	**[1.17**–**1.33]**			**1.24**	**[1.17**–**1.33]**
*Primary*			**1.45**	**[1.34**–**1.57]**			**1.44**	**[1.33**–**1.57]**
*No education*			**1.39**	**[1.29**–**1.51]**			**1.39**	**[1.29**–**1.51]**
***Wealth index***								
*Richest (Ref)*			1.00				1.00	
*Richer*			**1.09**	**[1.01**–**1.18]**			**1.11**	**[1.02**–**1.21]**
*Middle*			**1.16**	**[1.08**–**1.25]**			**1.20**	**[1.11**–**1.30]**
*Poorer*			**1.32**	**[1.23**–**1.43]**			**1.38**	**[1.27**–**1.50]**
*Poorest*			**1.46**	**[1.35**–**1.58]**			**1.53**	**[1.41**–**1.67]**
***Sex of child***								
*Male(Ref)*			1.00				1.00	
*Female*			**1.25**	**[1.20**–**1.30]**			**1.25**	**[1.20**–**1.30]**
***Birth Status***								
*Singleton(Ref)*			1.00				1.00	
*Multiple*			**11.88**	**[10.16**–**13.90]**			**11.89**	**[10.17**–**13.91]**
***ANC visit***								
*No (Ref)*			1.00				1.00	
*1 to 3*			0.92	[0.83–1.02]			0.92	[0.83–1.02]
*4 or above*			**0.83**	**[0.75**–**0.91]**			**0.83**	**[0.75**–**0.91]**
***Iron tablets/syrup intake during pregnancy***								
*Yes (Ref)*			1.00				1.00	
*No*			**1.14**	**[1.07**–**1.21]**			**1.14**	**[1.07**–**1.21]**
***Mode of birth***								
*Normal (Ref)*			1.00				1.00	
*Caesarean section*			**1.14**	**[1.09**–**1.20]**			**1.14**	**[1.08**–**1.19]**
**Community-level variables**								
***Residential Status***								
*Rural (Ref)*					1.00		1.00	
*Urban*					**0.89**	**[0.85**–**0.97]**	**1.10**	**[1.05**–**1.16]**
***Random Part***								
Community variance	0.59[0.56–0.62]		0.52 [0.50–0.69]		0.58 [0.56–0.62]		0.50 [0.49–0.65]	
ICC (%)	15.21%		13.65%		14.99%		13.20%	
*LR-test*	P < 0.001		P < 0.001		P < 0.001		P < 0.001	
*Log Likelihood*	−103165.14		−75538.51		−103154.26		−75521.48	
*AIC*	204946.6		149984.7		204905.4		149968.4	
*BIC*	204967.3		150165.5		204936.3		150159.3	

Abbreviation: ICC= Intra-class Correlation; LR-test = Likelihood Ratio test; AIC = Akaike information criterion; BIC= Bayesian information criterion. Bold indicates values are significant at a 1% level of significance.

a Intercept only model.

b Model includes only individual-level variables.

c Model includes only community-level variables.

d Model includes individual-level and community-level variables.

The variation in the intercept-only model (Model 0) was reduced by 11.86% after incorporating individual-level variables in Model 1. The full model (Model 4) explained 3.85% variations by including individual-level and community-level variables compared to Model 1. The model shifting indicates the variation across the clusters dropped from 15.21% (Model 0) to 13.20% (Model 3).

Women from the poorest quintile have 1.53 times (AOR: 1.53, 95% CI: 1.41–1.67) higher odds to deliver LBW babies than women from the wealthiest quintile. Uneducated women had a 39% (AOR: 1.39, 95% CI:1.29–1.51) higher odds of giving birth to LBW-afflicted babies than educated women. The number of increased ANC visits during pregnancy reduced the odds of being LBW among children by 0.83 folds (≥4 visits). Women who did not take iron supplements during pregnancy had a 14% (AOR: 1.14, 95% CI: 1.07–1.21) higher odds of having LBW children than those who took supplements.

Further, the findings illustrate that female children had 25% (AOR: 1.25, 95% CI: 1.20–1.30) higher odds of being LBW than male children. Besides, multiple births increased the odds of LBW by 11.89 folds (AOR: 11.89, 95% CI: 10.17–13.91) among children compared to singleton birth. The caesarean section increases the odds of LBW by 14% (AOR:1.14, 95% CI: 1.08–1.19) compared to normal delivery.

In the community-level variable, residential status significantly impacted instances of LBW. Women from urban areas have 1.10 times higher odds (AOR: 1.10, 95% CI: 1.05–1.16) to have LBW children than women from rural areas.

## 4 Discussion

Upon checking the currently available literature, this is the first study to investigate and measure SES inequalities in LBW among under-five children in South Asia, using the most contemporary nationally representative population-based data. The analysis provides a strong association between LBW and SES, showing women with lower SES having increased rates of LBW-afflicted children than women with higher SES across the South Asian subcontinent. The findings were corroborated by income and education-related inequalities in cases of LBW, arguing that poorer and less-educated women were more likely to have LBW babies. This coincides with the South Asian inequities policy report, which posits that South Asian countries have failed to achieve regional economic progress that benefits the poor and reduces pervasive inequality (Rama et al., 2015; South Asia Alliance for Poverty Eradication, 2019).

The results also illustrate how the magnitude and patterns of SES inequalities in LBW instances vary across South Asian countries. Both wealth and education-related inequalities in LBW were highly considerable in India, echoing a previous Indian study which revealed a significant association between SES inequalities and LBW (Mishra et al., 2021). In contrast, the disparities that appeared to be substantial in Nepal and Bangladesh seemed to be less pronounced. The education-related inequalities of LBW were apparent in Pakistan and Afghanistan but were found to be less potent in wealth-related inequalities. In the Maldives, however, LBW discrepancies in wealth and education were not as considerable. It suggests that children from India had the greatest SES disparities in LBW, followed by those from Nepal, Bangladesh, Afghanistan, and Pakistan. Hence, children from Afghanistan, India, Bangladesh, Nepal, and Pakistan made a significant contribution to South Asia's overall SES disparities. UNICEF's nutrition section claimed that maternal thinness, short stature, and anaemia had been associated with pregnancy abnormalities(Harding et al., 2018, Harding et al., 2018, United Nations Children’s Fund, 2019), were extensively observed in Afghanistan, India, Bangladesh, Nepal, and Pakistan, while those from the Maldives show lowered the prevalence of anaemia (United Nations Children’s Fund, 2019). This is a plausible justification for the result of the current study, as women's nutritional status before and during pregnancy significantly impacts their wellness and foetal growth (Kim et al., 2017; United Nations Children’s Fund, 2019). More scientific review on a large scale is required to determine the interaction between women's poor nutritional status and likelihood of LBW cases considering SES inequalities across countries in South Asia.

The current investigations of regional data demonstrate that maternal determinants have a significant role in LBWs. Maternal indicators, such as maternal age and education, considerably influenced LBW likelihood among South Asian children. Reduced maternal age and lower levels of education were associated with a substantial rise in LBWs, which lie consistent with prior studies (Abeywickrama et al., 2020; Khan et al., 2018; Mishra et al., 2021; Momeni et al., 2017; Tessema et al., 2021). Early childbearing is also linked to malnutrition or anaemia, which might trigger the birth of LBW children among younger women (Hampton, 2010; Tessema et al., 2021). Better education improves awareness about dietary patterns and quality of life behaviours throughout pregnancy (Tessema et al., 2021), which could be the plausible reason for the higher prevalence of LBWs in uneducated women compared to literate ones.

Pregnancy-related maternal health-seeking behaviours, such as increased ANC visits and iron supplementation, are associated with a significant reduction of LBW cases among South Asian children. These coincide with previous research conducted in the South Asian and Sub-Saharan African subcontinents, which suggested that one or more ANC visits were related to a decreased risk of LBW, compared to individuals who did not receive follow-ups (Dharmalingam et al., 2009; Khan et al., 2018; Mishra et al., 2021; Tessema et al., 2021). ANC visits enable ongoing monitoring during the prenatal period by providing nutritional education and recommending supplements for the mother and fetal health development. This may help deter unfavourable pregnancy complexities such as LBW (Tessema et al., 2021).

A comprehensive review established a strong link between iron supplementation and a decreased risk of LBW (Long et al., 2012), which corroborates the findings of the study. The same study also found inadequate evidence to notice the adverse impact of iron supplementation on birth outcomes, including instances of LBW. Iron supplementation improves hematologic iron status and protects against iron deficiency anaemia during maternity, reducing LBWs (Long et al., 2012). This disagrees with another study conducted in Sub-Saharan Africa which found no relationship between LBW and iron supplement intake (Tessema et al., 2021).

The current outcomes reveal that multiple birth or female gender has a significant adverse effect on birth weight; these findings are consistent with those of other studies (Khan et al., 2020; Mberu et al., 2016; Mishra et al., 2021; Tessema et al., 2021). Multiple births correlate with an increased need for nutrition; hence, this might cause LBWs (Tessema et al., 2021).

The study also illustrates that women who gave birth through caesarean section also have an increased risk of LBW compared to normal delivery. This coincides with WHO's LBW policy report stating how early inducement of labour or caesarean birth is a significant cause of LBW (World Health Organization, 2014). The decrease in wealth quintiles had a higher probability of having LBW children, echoing studies in developing and least developed countries (Abeywickrama et al., 2020; Gupta et al., 2019; Khan et al., 2018; Mishra et al., 2021). One probable explanation for this might be that early identification of fetal growth retardation becomes more prevalent among pregnant women as economic conditions improve (Tessema et al., 2021). Women from urban areas were more likely to have LBW children than women from rural areas, corroborating particular research from a worldwide setting (Gupta et al., 2019; Mishra et al., 2021). This finding contradicts Indian and Ghanaian investigations in which rural living was identified as a significant risk factor (Kayode et al., 2014; Metgud et al., 2012). However, Bangladeshi and Sri-Lankan studies observed no statistically significant association between geographic residence and LBWs (Abeywickrama et al., 2020; Khan et al., 2018). The possible explanation for these discrepancies could be methodological differences across the studies, as the study considered the clustering effect. Varying definitions of “urban” and “rural” might be the other supposed cause of this observed disparity.

The study's key novelty is the discovery of SES inequalities in LBW among South Asian children. Children afflicted with LBW are common among impoverished and illiterate women, particularly in Afghanistan, Bangladesh, India, Nepal, and Pakistan. To reduce the disparities in LBW, sophisticated initiatives based on knowledge of health education and poverty alleviation might be launched in South Asia, particularly in all the regions that are prone to SES inequalities. The WHO recommendation for ANC service utilisation during the prenatal period should be broadened to control the LBW prevalence (United Nations Children’s Fund, 2019, World Health Organization, 2014). This strategy incorporates various evidence-based tactics and nutritional knowledge, such as iron supplements. This should be directed at the poorest and least educated women in vulnerable territories (Afghanistan, Bangladesh, India, Nepal, and Pakistan). Considering the significance of birth history, geographical location, and gender in determining LBW in South Asia, policies should emphasise these dimensions. Thus, several NGOs and regional organisations should play a pivotal role in designing holistic strategies incorporating childcare and maternal health-related knowledge.

The current study utilised a nationally representative population-based sample in South Asia to assess LBW on an aggregate level. Estimating LBW inequities will assist health organisations and stakeholders in developing interventions to address this issue. The study's main strength is that it adopted three concentration indexes to assure the validity and precision of the estimates. The study also identified different South Asian countries' contributions to the overall SES inequalities in LBW. This study uses pooled effects to address associated factors, which might aid in generalising the impact.

The casual relationship cannot be drawn due to the cross-sectional design. The respondents' weight status was collected and subjected to recall bias. There is a lack of information on genetic factors, maternal clinical indicators, and consumption status of nutrition/dietary. The Body Mass Index was not included in the study because it was not accessible in the Afghanistan dataset.

## 5 Conclusion

The study depicts the SES inequalities in LBW cases among children under five in South Asia. In many countries in the subcontinent, most notably Afghanistan, Bangladesh, India, Nepal, and Pakistan, women of lower SES, *i.e.* those from the poorest households and with less education, are more likely to have LBW children. Governments and NGOs of South Asia might need to focus on health education and knowledge of poverty alleviation to decrease SES inequalities in LBW. Factors such as maternal age and education, wealth status, sex of the child, birth status, number of ANC visits, iron supplementation, mode of birth, and residential status were all significant indicators of LBW children in South Asia. Public health initiatives targeting maternal health should address these significant indicators to minimise the occurrence of LBWs, with a particular emphasis on women from Afghanistan, Bangladesh, India, Nepal, and Pakistan and those with lower SES levels. Expanding the coverage and raising public awareness of the ANC program and iron supplement requirements during pregnancy are also essential. Increasing awareness of health education concerning delivery complications, including LBW, is required to reduce the LBW prevalence. These recommendations will aid in reaching the WHO's nutrition targets by ensuring the normal birth weight of children, directly assisting in attaining the connected SDGs in the future.

## Ethics approval and consent to participate

The data is freely available at http://www.dhsprogram.com and does not contain any personally identifiable information. The authors obtain access to data after procuring authorisation via an online application. Each parent or guardian was supplied with an informed consent statement prior to the interview. It guarantees the legal participation and secrecy of each child.

## Consent for publication

Not applicable.

## Availability of data and materials

The data utilised in the study was acquired through the program Demographic and Health Survey. Data are available at http://www.dhsprogram.com. Any subsequent requests for additional inquiries can be referred to the corresponding author.

## Funding

This research did not receive any specific grant from funding agencies in the public, commercial or not-for-profit sectors.

## Authors' contributions

NJS, BA, and SAK initiated the study, and conducted the data analysis. NJS, BA, and SAK drafted the manuscript. KA, RH, and KYL offered advice, critical comments and edited the draft manuscript. All the authors read and approved the final manuscript.

We wish to confirm that there are no known conflicts of interest associated with this publication and there has been no significant financial support for this work that could have influenced its outcome.

We confirm that the manuscript has been read and approved by all named authors and that there are no other persons who satisfied the criteria for authorship but are not listed.

We further confirm that the order of authors listed in the manuscript has been approved by all of us.

We confirm that we have given due consideration to the protection of intellectual property associated with this work and that there are no impediments to publication, including the timing of publication, with respect to intellectual property. In so doing we confirm that we have followed the regulations of our institutions concerning intellectual property.

We further confirm that any aspect of the work covered in this manuscript that has involved either experimental animals or human patients has been conducted with the ethical approval of all relevant bodies and that such approvals are acknowledged within the manuscript.

## Declaration of competing interest

The authors have no conﬂicts of interest to declare.

## Data Availability

Data will be made available on request.

## Abbreviations

WHO: World Health Organization
LBW: Low birth weight
SES: Socioeconomic status
DHS: Demographic and Health Survey
LMICs: Low-and middle-income countries
AOR: Adjusted Odds Ratio
SDGs: Sustainable Development Goals
ANC: Antenatal Care
MOR: Median Odds Ratio
