# Supplementary material for: Socioeconomic inequalities in low birth weight in South Asia: A comparative analysis using Demographic and Health Surveys
Source: SSM Popul Health. 2022 Oct 11;20:101248. doi: 10.1016/j.ssmph.2022.101248 (PMC9587321; doi:10.1016/j.ssmph.2022.101248)
Supplement: Multimedia component 1 [file mmc1.docx]

**Additional file 1: Concentration curves drawn by Wagstaff and Erreygers concentration index**


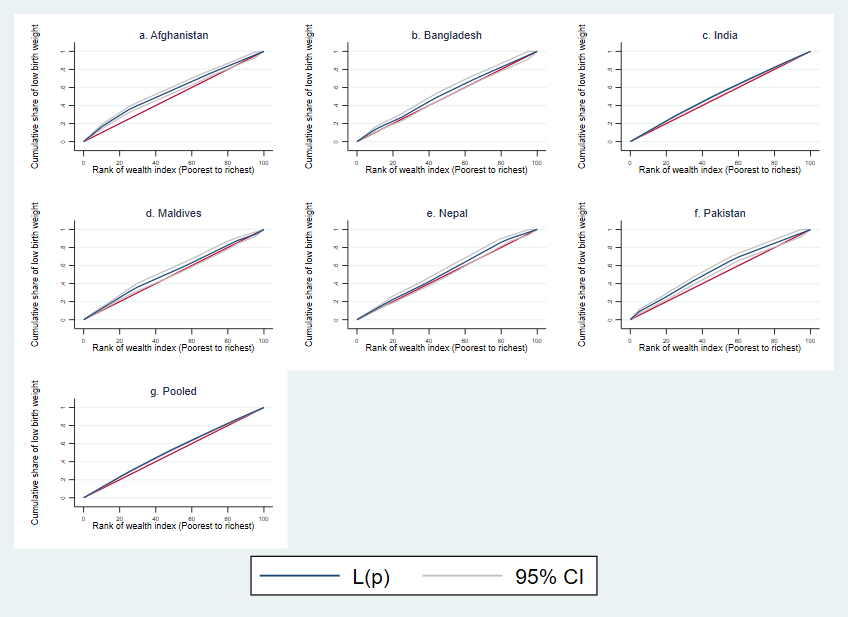


**A1** Concentration curve of low birth weight (by Wagstaff concentration index with wealth index ranking)


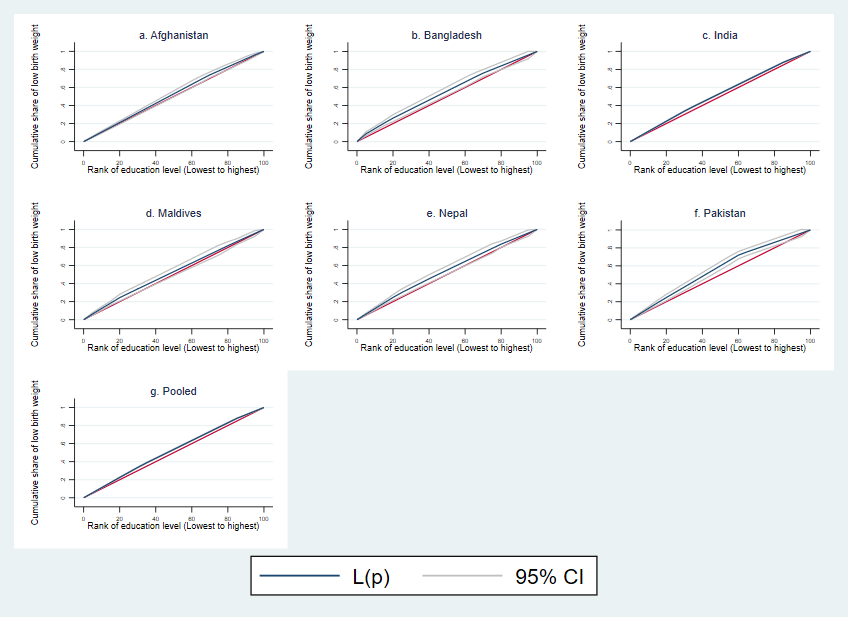


**A2** Concentration curve of low birth weight (by Wagstaff concentration index with education level)


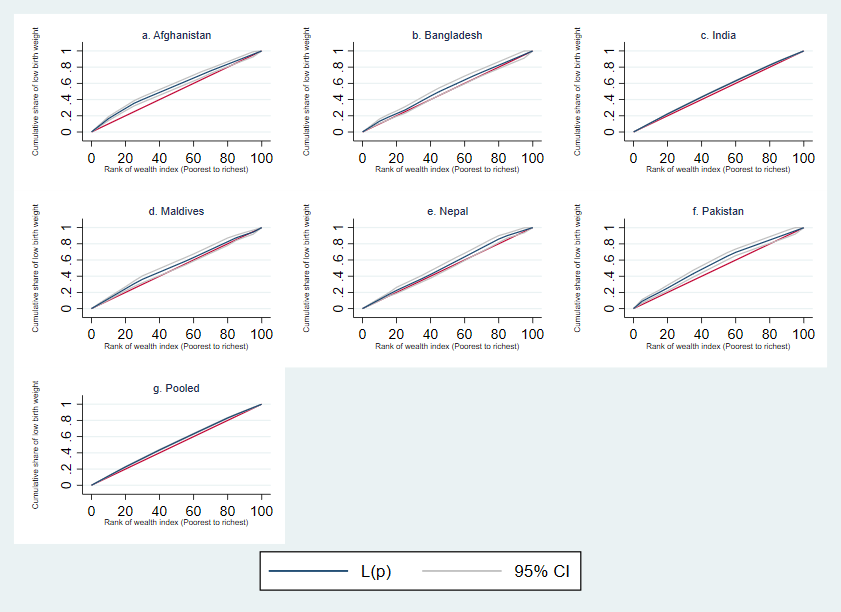


**A3** Concentration curve of low birth weight (by Erreygers concentration index with wealth index ranking)


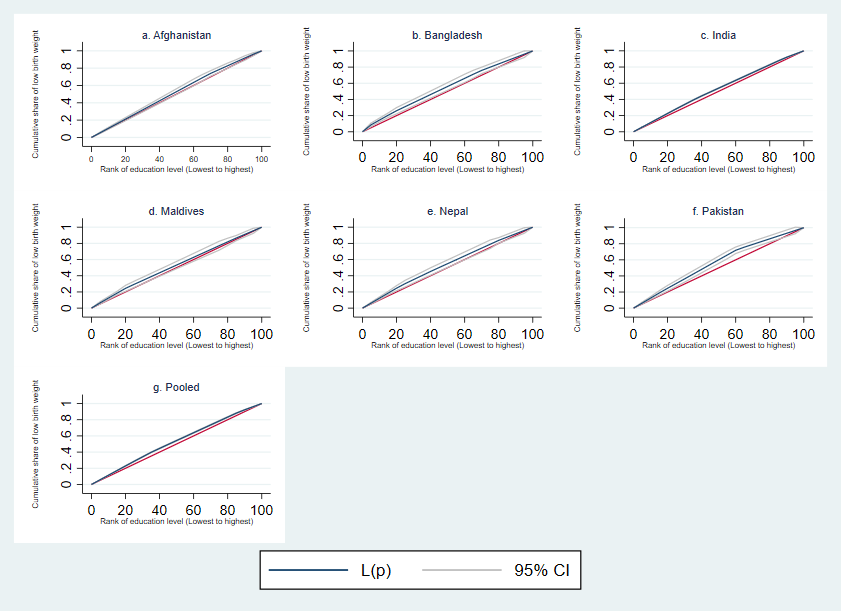


**A4** Concentration curve of low birth weight (by Erreygers concentration index with education level)
